# Recent insights into the role of hormones during development and their functional regulation

**DOI:** 10.3389/fendo.2024.1340432

**Published:** 2024-01-22

**Authors:** Youssef Aref, Shelby Chun Fat, Edward Ray

**Affiliations:** Cedars-Sinai Medical Center, Department of Surgery, Division of Plastic and Reconstructive Surgery, Los Angeles, CA, United States

**Keywords:** hormone, development, regulation, puberty, functional regulation

## Abstract

**Introduction:**

Hormones play a vital role in development from conception to birth and throughout the human lifespan. These periods are logically divided into fetal development, pre-pubertal growth, puberty, and adulthood. Deviations from standard physiological levels and release patterns of constituent hormones can lead to pathology affecting the normal developmental trajectory. Research is ongoing to better understand the mechanisms of these hormones and how their modulation affects development.

**Methods:**

This article focuses on recent developments in understanding the role hormones play in development. We also cover recent discoveries in signaling pathways and hormonal regulation.

**Results:**

New and continuing research into functional hormone regulation focuses on sex hormones, gonadotropic hormones, growth hormones, insulin-like growth factor, thyroid hormone, and the interconnectedness of each of these functional axes. Currently, the abundance of work focuses on fertility and correction of sex hormone levels based on an individual’s condition and stage in life.

**Discussion:**

Continuing research is needed to fully understand the long-term effects of hormone modulation in growth and sexual development. The role of each hormone in parallel endocrine axes should also be more thoroughly investigated to help improve the safety and efficacy in endocrine pharmacotherapeutics.

## 1 Introduction

### 1.1 Overview of hormones in development

Human development is the process of physical and mental growth from conception throughout the life of the person. From conception, hormones guide the formation and regression of structures for somatic and reproductive development (1). Between birth and adolescence, hormones from the anterior pituitary gland, such as growth hormone (GH), adrenocorticotropic hormone (ACTH), thyroid hormone (TH), follicle-stimulating hormone (FSH), and luteinizing hormone (LH), each play a specific role in the progression of normal anatomy and physiology (2, 3). During puberty, the levels of hormones affecting growth and reproductive organs drastically change for an intense period of maturation (4). Following puberty, the function of these hormones transition from promoting development to maintaining normal physiology. Abnormalities in the levels or release of any of these hormones can lead to pathologies affecting metabolism and growth, which vary depending on the time of life during which these irregularities manifest (5). Therapeutically modulating the levels of each hormone is an expanding field of research, with more precise and effective methods under investigation. This article describes current knowledge about the role of hormones in development and contemporary research into functional hormone regulation. The areas of focus described here include early development (before puberty), pubertal development, and hormonal contributions during adult life.

## 2 Hormones before puberty

### 2.1 Role of hormones in early development

#### 2.1.1 Fetal development

Fetal development is shaped by the interplay of endogenous hormone production and the maternal endocrine system. During early fetal growth, the endocrine glands are being formed and until these organs function independently, the fetus relies upon the mother’s physiology. The placenta is the sine qua non endocrine organ supporting fetal growth through production of peptide and steroid hormones, each with its own mechanism. Human chorionic somatomammotropin (hCS), formerly referred to as human placental lactogen (hPL), increases delivery of glucose to the fetus by down-regulating maternal utilization of glucose and by stimulating fatty acid metabolism. hCS’s exact role in pregnancy outcomes is a topic of ongoing study with current evidence suggesting its association with placental mass and infant birthweight (6). Due to its critical role, hCS is used as a marker for the effects of glucose transporters during fetal development (7).

Growth hormone (GH) as well as human GH variant (hGH-V) support fetal growth through the actions of insulin-like growth factors. Insulin-like growth factors (IGF) 1, 2, and 3 each utilize their respective receptors and IGF-binding proteins to stimulate cell proliferation, survival, and growth (8, 9). IGF-1 is expressed across a variety of fetal tissues with highest expression in the lung and intestine while IGF-2 is mainly expressed in the fetal kidney, liver, adrenal glands, and muscle (10). IGF-1 has recently been identified as a key product of the glutamine metabolic pathway of decidual NK cells, promoting trophoblast invasion to prevent pregnancy loss (11).

Glucocorticoids and sex steroids also have their function during this early stage of life. Cortisol is recognized as vital for proper fetal development through its negative feedback effects on the hypothalamic-pituitary-adrenal (HPA) axis while also regulating adrenal steroid hormone production (12).

Anti-Müllerian hormone (AMH) plays a specialized role in fetal sexual development. *In utero*, this hormone causes regression of the Müllerian ducts which would otherwise form female reproductive structures. AMH belongs to the transforming growth factor beta (TGFB) family, specifically acting on type II serine/threonine kinase receptors with additional activity through type I receptors on bone. Absence of either AMH or its receptors can prevent regression of female reproductive structures and thus affect sexual differentiation (13). AMH is currently being studied for its association with polycystic ovarian syndrome (PCOS) (14). Levels of this hormone are significantly higher in women with PCOS compared to those without PCOS, providing an alternative to ultrasound in diagnosing this condition (15). During fetal development, AMH has also been implicated as a potential cause of spontaneous abortion by inhibiting placental aromatase and, in turn, increasing fetal exposure to estradiol and progesterone (16). Current studies are investigating the importance of AMH in predicting live birth to resolve inconsistencies in the literature (17). Ongoing research is examining the influence of AMH in sexual differentiation disorders, as a tumor marker for granulosa cell tumors, as well as its potential utility in treating gynecological malignancies that express AMH-specific receptors (18).

Estrogen is produced by the placenta, using dehydroepiandrosterone sulfate (DHEAS) from the fetal adrenal glands as its substrate (8). Additionally, glucocorticoids are vital in regulating fetal organ development and maturation. The placenta regulates diffusion of cortisol from the mother to the fetus, preventing excessive exposure through production of 11-beta-hydroxy-steroid enzymes. Research suggests that the ratio of these enzymes affects the maturation of the fetal hypothalamic-pituitary-adrenal axis (19).

Outside of steroid hormones, maternal TH plays a critical function in fetal neural development. Children of mothers with decreased TH levels have higher rates of neurologic pathology such as congenital iodine deficiency syndrome, formerly referred to as cretinism (20). These effects are likely modulated through a variety of receptors and intracellular pathways that are actively being studied to better understand this aspect of fetal development (21).

#### 2.1.2 Childhood growth

The main hormonal interactions during childhood and prior to puberty come from GH/IGF, TH, and gonadal steroids (22). GH, as the name suggests, stimulates growth during childhood. GH deficiency can lead to short stature and when recognized early, is treatable with GH injections. While GH replacement is safe and a standard-of-care treatment, the timing and hormone formulation (short vs long-acting GH) is currently under study with the aim of limiting the burden frequent injections can place on families (23–25).

TH plays an important role in metabolism and can cause downstream effects on growth, as well as pubertal and intellectual development (26). Disorders of TH levels are typically due to autoimmune conditions and iodine deficiency (27, 28). Just as with GH supplementation, deficiencies of iodine are treated with iodine supplementation (29). Recent research into developmental thyroid disorders include uncovering potential mechanisms by which stress alters the hypothalamic-pituitary-thyroid (HPT) axis as a source of inflammation that leads to thyroid cancer (30).

Adrenal hormones also play a significant role in pediatric development. Congenital adrenal hyperplasia (CAH) includes several genetic abnormalities of adrenal steroid hormone production with resulting effects on sexual development prior to puberty (31). The most recommended CAH treatment, though controversial for its potentially adverse effects on cognitive development, metabolism, and risk of orofacial cleft, is prenatal dexamethasone treatment to prevent excessive levels of androgens that typically cause virilization in patients (31). Investigations into prenatal dexamethasone administration are ongoing to determine long-term outcomes and risk-ratio profile. Its efficacy and benefits appear to depend in part on parental genotype (32, 33). The use of hydrocortisone and fludrocortisone have also been shown as safe treatments for some forms of CAH, such as 11-beta hydroxylase deficiency (34). Novel CAH treatments have focused on alternative hypothalamic-pituitary-adrenal axis targets and the timing of drug delivery. New areas for research include the investigation of corticotropin-releasing factor (CRF) antagonists (35) and abiraterone acetate, a CYP17A1 inhibitor, to treat androgen excess in CAH (36).

## 3 Hormones during puberty

During adolescence, there is a significant change in the levels of hormones, particularly gonadotropic releasing hormone (GnRH), FSH and LH, as well sex gonadal sex steroids that lead to sexual development (37). Certain hormonal developments are unique to sex, such as mammary gland development, which accelerates during puberty. GH, IGF-1, and estrogen are thought to be the most important factors in pubertal mammary development, acting to support ductal growth (38). While puberty usually occurs at set times for males and females, precocious and delayed puberty inspired the field of research that investigates the causes and treatments for abnormal sex hormone levels. Recently, the gut microbiome has been a focus of metabolic research due to its role in health and homeostasis, intersecting several bodily systems. The microbiota, largely passed on from mother to child, has been shown to influence both metabolism and the production of certain hormones. Work on causes and treatment of precious puberty, for example, has examined the role of microorganisms (39).

With increasing attention paid to gender dysphoria and transgender health, there has been a rise in research on hormone modulation, including puberty blockers and their long-term sequelae. Mouse experiments using prepubertal administration of leuprolide acetate (LA) and testosterone found similar long-term outcomes in ovarian function and embryologic development when compared with controls, suggesting no permanent reproductive impairment with puberty blockers (40). However, current recommendations, based on studies on the effects of puberty blockers in humans, are to delay puberty blockade until after germ cell maturation to preserve future fertility. Fertility preservation has been most successful in transgender males (i.e., individuals assigned female at birth but who identify as male) likely because they are born with all the germ cells they will ever have. Puberty blocker administration has no effect on germ cell maturation in these individuals (41). Histrelin (GnRH agonist) implants act as puberty blockers by continuously eluding this drug. These implants have been shown to be a safe treatment for both precocious puberty and gender dysphoria (42).

## 4 Hormones after puberty

After puberty, there is a period of continued somatic growth, after which the endocrine system has fully matured. Hormones then mainly serve the purposes of regulating adult metabolism and maintaining fertility.

Sex steroid hormones (SSH) are being studied for their role in treating certain depressive disorders in adults. While the mechanisms are still under investigation, future studies will examine the downstream effects of targeting GABA and other receptor signaling pathways across genders, ages, and health conditions (43). SSHs are also being assessed for their role in other body systems. One study found, for example, that while estradiol is not associated with periodontitis, sex hormone-binding globulin (SHBG), testosterone, and free androgen levels were negatively associated with periodontitis and even more strongly in individuals under 50 (44).

In the adult musculoskeletal system, hormones help maintain bone and soft tissue integrity. The sources of GH and IGF in the adult skeleton are still under investigation, though bone marrow stromal cells (BMSCs) are one source of IGF known to support osteogenesis while inhibiting adipogenesis in the adult skeleton (45). Growth hormone has also been explored for its role in other endocrine axes, including fertility research for its potential to improve IVF outcomes (46).

With an increasing number of transgender and gender non-binary individuals being prescribed long-term exogenous hormones, the prolonged effects of sex hormones on mature hormone-sensitive tissues is gradually coming to light. Testosterone, for example, induces cellular changes that alter glandular tissue density and fat distribution in the mature breast, leading to a decrease in breast size and modified ductal morphology (47). The implications for breast cancer risk in transgender men are unknown and studies on this subject have been largely inconclusive, due in part to small sample sizes. One systematic review found a slightly higher incidence of breast cancer after testosterone use in transgender men compared to cisgender men while others have found no such relationship (48, 49). There is evidence that elevated serum androgen levels are linked to higher breast cancer risk in post-menopausal women, and it is known that androgen receptor (AR) blockade may help treat some AR-positive breast cancers by inhibiting tumor growth, though this observed behavior varies with breast cancer subtype (50–52). Other areas of investigation specific to transgender individuals include the lifetime risk of osteoporosis, cardiovascular disease and non-breast glandular (e.g., prostate) cancers under the influence of prolonged exogenous hormone therapy (53, 54).

## 5 Discussion

Hormones each play a unique and important role in development at different stages of life. During fetal development, placental hormones take main stage to support nutrient delivery, fetal growth, and maintain healthy hormone levels until fetal structures develop sufficiently to assume this role. Prior to puberty, development centers around proper musculoskeletal, neurologic, and metabolic development. During puberty, sex steroids along the hypothalamic-pituitary-gonadal axis support reproductive maturation. Following puberty into adulthood, hormones are most important for maintaining metabolism, musculoskeletal health, and fertility.

Research into each of these phases aims at understanding the role of hormones and interplay within, and across, their respective axes. This also includes better understanding how to restore physiologic levels of hormones in the midst of pathology. Hormone modulation is also critical in the treatment of conditions ranging from delayed puberty to gender dysphoria (39–41).

## 6 Conclusion

While research is ongoing to gain a more comprehensive understanding of all hormones involved in development, the greatest focus continues to be on sex hormones. This may be due to their role throughout the lifespan, from sexual development to fertility after puberty. The increase in attention to transgender and gender non-binary health also has sparked more investigation into safe and effective hormonal therapeutics. Continued work is needed to more completely piece together the mechanisms and interplay of hormonal axes and how these impact human development and health.

## Author contributions

YA: Writing – original draft, Writing – review & editing. SF: Conceptualization, Writing – original draft. ER: Conceptualization, Project administration, Writing – original draft, Writing – review & editing.
